# Triglyceride glucose index, a marker of insulin resistance, is associated with coronary artery stenosis in asymptomatic subjects with type 2 diabetes

**DOI:** 10.1186/s12944-016-0324-2

**Published:** 2016-09-15

**Authors:** Eun Young Lee, Hae Kyung Yang, Joonyub Lee, Borami Kang, Yeoree Yang, Seung-Hwan Lee, Seung-Hyun Ko, Yu-Bae Ahn, Bong Yun Cha, Kun-Ho Yoon, Jae Hyoung Cho

**Affiliations:** 1 Division of Endocrinology and Metabolism, Department of Internal Medicine, Seoul St. Mary’s Hospital, College of Medicine, The Catholic University of Korea, 222, Banpo-daero, Seocho-gu, Seoul, 06591 Republic of Korea; 2 Division of Endocrinology and Metabolism, Department of Internal Medicine, St. Vincent’s Hospital, College of Medicine, The Catholic University of Korea, Seoul, Korea; 3 Institute of Catholic Ubiquitous Health Care, The Catholic University of Korea, Seoul, Korea

**Keywords:** Atherosclerosis, Coronary, TyG index, Type 2 diabetes

## Abstract

**Background:**

Insulin resistance is one of the most important contributing factors to cardiovascular disease. This study aimed to investigate the association between coronary artery stenosis (CAS) and triglyceride glucose index (TyG index), a simple insulin resistance marker, in asymptomatic subjects with type 2 diabetes.

**Methods:**

We recruited asymptomatic adults with type 2 diabetes but without previous history of coronary heart disease (*n* = 888). Significant CAS was defined as maximum intraluminal stenosis ≥70 % by coronary CT angiography. TyG index was calculated as log [fasting triglycerides (mg/dl) x fasting glucose (mg/dl)/2].

**Results:**

Mean age was 63.8 ± 9.5 and 58.9 % of the subjects were men. We analyzed the participants according to the tertile of TyG index. The TyG index was correlated with HOMA-IR (*r* = 0.397, *P* < 0.001), and subjects with higher tertile of TyG index were younger but showed worse clinical and metabolic parameters. The prevalence of CAS was higher in subjects with higher tertile of TyG compared with those with lower tertile of TyG (14 % vs. 7.8 %, *P* = 0.022). On multiple regression analysis, the highest tertile of TyG index was an independent risk factor for CAS after adjustment for other confounders (odds ratio, 3.19 [95 % CI, 1.371–7.424]). Subgroup analysis showed that TyG index showed more significant association with CAS in patients with risk factors such as old age, longer duration of diabetes, poor glycemic control, no statin use, and male gender.

**Conclusion:**

Higher TyG index is associated with increased risk of CAS in asymptomatic subjects with type 2 diabetes, particularly when they have risk factors for cardiovascular disease.

**Trial registration:**

This study was retrospectively registered in ClinicalTrials. gov with the registration number of NCT02070926 in Feb 23, 2014.

## Background

Cardiovascular disease (CVD) is a major concern in subjects with type 2 diabetes as it ultimately can lead to severe morbidity and mortality. In type 2 diabetes, macrovascular complications are the primary cause of mortality, accounting for more than 50 % of all death [1]. In addition to the high prevalence of CVD in type 2 diabetes, the recurrence rate of CVD is very high (around 6 %/year) in subjects with type 2 diabetes and prior CVD [2, 3]. In Korea, the prevalence of ischemic heart disease is about 4 times greater in patients with diabetes compared to those without diabetes [4]. Furthermore, coronary intervention or bypass surgery was performed at frequencies of up to 10-fold higher in patients with type 2 diabetes than those without diabetes [4]. Therefore, it is important to identify subjects at high risk for CVD and to manage their condition early, particularly in those with type 2 diabetes.

One of the most important contributing factors to CVD is insulin resistance, which refers to a decreased sensitivity and responsiveness to the metabolic actions of insulin. Insulin resistance predisposes to several disorders such as hyperglycemia, hypertension, and dyslipidemia, all of which are strongly associated with atherosclerosis. In addition, insulin resistance contributes to vasoconstriction, inflammation, and thrombosis, leading to accelerated atherosclerosis. Previous studies have demonstrated an independent association between insulin resistance and clinical or subclinical CVD in both non-diabetic and diabetic subjects [5–8]. In non-diabetic subjects, insulin resistance is associated with the presence of coronary artery diseases and incident CVD [6, 7]. In diabetic subjects, insulin resistance is associated with the thickening of the carotid artery intima-media and incident CVD [5, 8].

Recently, a simple and inexpensive approach to evaluate for insulin resistance has been developed [9]. This particular test is based on measuring the product of fasting triglycerides and glucose. Therefore, we aimed to investigate the association between CAS and triglyceride glucose index (TyG index) in asymptomatic subjects with type 2 diabetes. To the best of our knowledge, this is the first study to investigate the association between TyG index and CAD.

## Methods

### Study subjects and design

This study was an observational cohort study based on the CRONOS-ADM Registry (Coronary CT angiography evaluation for clinical outcomes in asymptomatic patients with type 2 diabetes mellitus, registered with ClinicalTrials.gov NCT02070926). We recruited asymptomatic subjects with type 2 diabetes who underwent coronary CT angiography between Jan 2006 and Dec 2010. In the present study, we included subjects who were aged ≥18 years and had neither a history of ischemic heart disease nor symptoms of angina. We excluded those with type 1 diabetes, cardiac transplantation, ventricular or supraventricular arrhythmias, and ischemic heart disease such as coronary artery disease, myocardial infarction, and coronary revascularization. Subjects who had elevated triglyceride levels (>500 mg/dl) or had taken antianginal medication or medications lowering primarily triglycerides (fenofibrate, omega-3) were also excluded. We measured blood glucose, fasting insulin, and lipid profiles. HOMA-IR was calculated as insulin (mU/l) × (glucose [mg/dl] × 0.055)/22.5 [10]. The TyG index was calculated by the logarithm of fasting triglyceride x fasting glucose/2 [9]. Metabolic syndrome was assessed in those whose waist circumference data were available (*n* = 280). Metabolic syndrome was defined according to the revised criteria of the National Cholesterol Education Program Adult Treatment panel III (NCEP-ATP III) [11]. This study protocol was conducted in full compliance with the Declaration of Helsinki and was approved by the institutional review board of Seoul St. Mary’s Hospital, The Catholic University of Korea (No. KC15RISI0955). The written informed consent from the participants was waived by the institutional review board as only de-identified data were accessed and analyzed.

### Imaging protocol and analysis

Coronary CT angiography imaging was obtained using a 64-slice, dual source CT scanner (SOMATOM Definition, Siemens, Forchheim, Germany). All patients were in normal sinus rhythm and if the heart rate was faster than 70 beats per minute (bpm), intravenous beta blocker (esmolol) was administered at a dose of 3 mg at 5-min intervals up to three times. Images were obtained before and after administration of 80–110 mL of iodinated contrast (Lomeron 350, iomeprol, Bracco, Milano, Italy). All scans were performed using ECG-controlled tube current modulation and the estimated radiation dose was less than 14 mSv. To obtain coronary artery images, all images were reconstructed immediately after scan completion.

Coronary CT angiography images were analyzed by two experienced radiologists. According to the guidelines of the Society of Cardiovascular Computed Tomography [12], each segment of the coronary artery was scored visually for the presence of a coronary plaque using a 16-segment coronary artery model in an intent-to-diagnose fashion. All analysis was performed only on segments with a diameter ≥1.5 mm. Coronary plaques were defined as structures ≥1 mm^2^ within or adjacent to the coronary artery lumen, which were characterized from the vessel lumen or surrounding pericardial tissues. Significant CAS was diagnosed when there was severe coronary artery stenosis, or when the maximum intra-luminal stenosis in any of the segments of the major epicardial coronary arteries was greater than 70 % [13].

### Statistical analysis

Data are presented as mean ± standard deviations and number (percentages). ANOVA and post hoc analysis were performed to compare groups according to tertiles of TyG index. For nonparametric variables, data are presented as median (interquartile range) and the Kruskal-Wallis test was performed to compare three groups. Categorical variables were analyzed using the χ^2^ or Mantel-Haenszel χ^2^ test. In order to adjust for age and sex, partial Pearson’s correlation coefficient (R) was used to assess the relationships between the two variables. Multivariate logistic regression analysis was performed in order to control for confounding factors. Subgroup analysis was performed after categorizing the subjects according to age, sex, duration of diabetes, glycemic control, hypertension, and statin use. In subgroup analysis, odds ratio was compared between the highest and lowest tertile of TyG index. All statistical analyses were performed using SPSS (version 20.0; IBM SPSS, NY, USA). A *P* value <0.05 was considered statistical significant.

## Results

### Baseline characteristics according to the tertiles of TyG index

Mean age was 63.9 ± 9.5 years and 58.9 % (*n* = 523) of the study population (*n* = 888) were male. We analyzed participants according to tertiles of TyG index. Subjects in the higher tertile of TyG index were younger but had greater BMI, WC, WHR, and blood pressure (Table 1). Glycemic indices, such as plasma glucose and glycated hemobgloin, and serum uric acid levels were higher amongst subjects with higher tertile of TyG index. In addition, subjects with higher tertile of TyG index showed higher total and LDL-cholesterol levels but lower HDL-cholesterol levels. There were no differences in the duration of diabetes and renal function according to the tertiles of TyG index. In subjects with higher tertile of TyG index, more proportion used insulin and metformin while α-glucosidase inhibitor and antiplatelet agent use were less common. There was no difference in statin use according to the tertiles of TyG index. At baseline electrocardiography (ECG), 62 participants showed left ventricular hypertrophy (*n* = 62, 7.0 %). Other frequently observed changes in ECG were as follows: first degree atrioventricular block (*n* = 37, 4.2 %), right bundle branch block (*n* = 29, 3.3 %), atrial or ventricular premature complex (*n* = 22, 2.5), atrial fibrillation (*n* = 3, 0.3 %), T wave inversion (*n* = 3, 0.3 %), ST depression (*n* = 2, 0.2 %), and Q wave (*n* = 1, 0.1 %). The frequencies of these ECG changes were not different across the tertile of TyG index.

**Table 1:** Baseline characteristics of study population according to the tertiles of TyG index

	Lowest tertile (*n* = 293)	Mid tertile (*n* = 296)	Highest tertile (*n* = 299)	*P* value
Age (year)	65.4 ± 8.6	64.2 ± 9.3	62.0 ± 10.2*†	<0.001
Male (%)	180 (61.4)	172 (58.1)	171 (57.2)	0.295
Duration of diabetes (year)	12.3 ± 9.3	12.2 ± 9.6	11.8 ± 9.3	0.779
BMI (kg/m^2^)	23.2 ± 3.1	24.6 ± 3.0*	24.9 ± 3.3*	<0.001
WC (cm)	85.6 ± 8.5	89.8 ± 11.3*	89.8 ± 8.5*	0.005
WHR (cm)	0.9 ± 0.1	0.9 ± 0.1	1.0 ± 0.1*	0.010
SBP (mmHg)	123.7 ± 14.5	126.6 ± 13.3	127.0 ± 15.8*	0.016
DBP (mmHg)	73.2 ± 9.7	74.8 ± 9.5	77.0 ± 9.7*†	<0.001
TyG index	8.2 ± 0.3	8.9 ± 0.2*	9.7 ± 0.4*†	<0.001
HOMA-IR	2.1 (1.3–3.7)	3.9 (2.1–7.1) *	5.2 (3.2–10.1) *†	<0.001
FPG (mg/dL)	117.1 ± 28.8	138.8 ± 31.6*	188.4 ± 71.1*†	<0.001
PPG (mg/dL)	185.6 ± 65.8	205.6 ± 76.0*	245.6 ± 83.6*†	<0.001
HbA1c (%)	7.4 ± 1.8	7.7 ± 1.9	8.8 ± 2.0*†	<0.001
Total cholesterol (mg/dL)	155.5 ± 30.7	165.9 ± 32.8*	182.9 ± 42.5*†	<0.001
Triglyceride (mg/dL)	68 (54–83)	115 (94–138)*	188 (145–253) *†	<0.001
HDL cholesterol (mg/dL)	52.0 ± 13.4	46.5 ± 10.2*	44.2 ± 10.4*	<0.001
LDL cholesterol (mg/dL)	89.9 ± 27.4	96.1 ± 29.7	97.4 ± 37.7*	0.009
Estimated GFR (mL/min/1.73 m^2^)	95.7 ± 24.6	93.8 ± 25.1	93.1 ± 24.2	0.125
Uric acid (mg/dL)	4.9 ± 1.3	5.0 ± 1.4	5.2 ± 1.6	0.046
Insulin, n (%)	59 (20.1)	66 (22.3)	83 (27.8)	0.028
Sulfonylurea, n (%)	117 (39.9)	127 (42.9)	109 (36.5)	0.383
Meglitinide, n (%)	39 (13.3)	33 (11.1)	35 (11.7)	0.551
Metformin, n (%)	161 (54.9)	193 (65.3)	202 (67.6)	0.002
Thiazolidinedione, n (%)	8 (2.7)	8 (2.7)	12 (4.0)	0.370
α-glucosidase inhibitor, n (%)	69 (23.5)	63 (21.3)	36 (12.0)	<0.001
DPP-IV inhibitor, n (%)	19 (6.5)	25 (8.4)	22 (7.4)	0.689
Antiplatelet agent, n (%)	192 (66.2)	173 (58.6)	153 (51.7)	<0.001
Statin, n (%)	151 (51.5)	176 (59.5)	138 (53.8)	0.580

Data are expressed as mean ± SD, median (interquartile range), or N (%). ACR, albumin to creatinine ratio; *BMI* body mass index, *DBP* diastolic blood pressure, *DPP-IV* dipeptidyl peptidase IV, *FPG* fasting plasma glucose, *GFR* glomerular filtration rate, *HDL* high density lipoprotein, *HOMA-IR* homeostasis model assessment – insulin resistance, *LDL* low density lipoprotein, *PPG* postprandial plasma glucose, *SBP* systolic blood pressure, *WC* waist circumference, *WHR* waist to hip ratio. * vs. lowest tertile, † vs. mid tertile

### Association of TyG index with HOMA-IR and metabolic syndrome

**Fig. 1:**
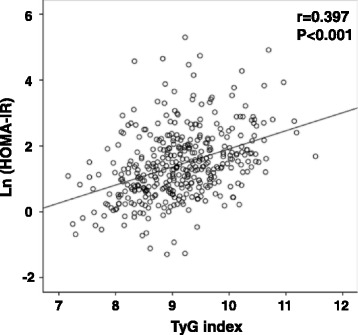
Correlation between TyG index and HOMA-IR after adjustment for age and sex

HOMA-IR values increased significantly according to the tertiles of TyG index (Table 1). After adjusting for age and sex, the TyG index was significantly correlated with the HOMA-index (Fig. 1). The prevalence of metabolic syndrome was significantly higher in subjects with the highest tertile of TyG index compared to subjects of the lowest tertile (84.1 % vs 39.4 %, *P* < 0.001). In addition, features of metabolic syndrome, such as hypertension, abdominal obesity, or low HDL-cholesterol were more common amongst subjects with the higher tertiles of TyG index (all *P* < 0.05).

### Association of TyG index with CAS

**Fig. 2:**
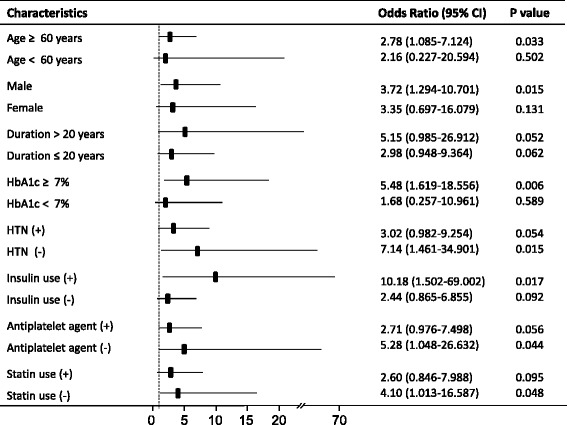
Subgroup analysis for coronary artery stenosis. Odds ratio of the highest tertile of TyG index was presented in comparison with the odds ratio of the lowest tertile of TyG index

Of the 888 subjects with asymptomatic type 2 diabetes, 109 (12.3 %) were found to have significant CAS on MDCT. The prevalence of significant CAS was higher in subjects with the higher tertile of TyG index (14 % for the highest tertile of TyG index vs 7.8 % for the lowest tertile of TyG index, *P* = 0.022). On multiple regression analysis (Table 2), the higher tertile of TyG index was significantly associated with CAS after adjusting for age and sex (odds ratio, 2.36 [95 % GI, 1.370–4.078] for mid tertile and 2.50 [1.432–4.348] for the highest tertile of TyG index). After further adjustment for other confounding factors, such as glycemic control, blood pressure, LDL-cholesterol and medications, the highest tertile of TyG index was an independent risk factor for CAS (odds ratio, 3.19 [95 % CI, 1.371–7.424]). As shown in Fig. 2, subgroup analysis showed that subjects aged ≥60 years and with the highest tertile of TyG index had an independent association with CAS (odds ratio, 2.78 [95 % CI, 1.085–7.124]). In addition, subjects with male gender, longer duration of diabetes, and poor glycemic control had higher odds ratios than those without these risk factors. Subjects receiving insulin also showed higher odds ratio compared to those without insulin therapy. Meanwhile, subjects receiving antiplatelet agent or statin had lower odds ratios compared to those without these medications.

**Table 2:** Multiple regression analysis for coronary artery stenosis

	Model 1		Model 2		Model 3		Model 4	
TyG index tertile	OR (95 % CI)	*P* value	OR (95 % CI)	*P* value	OR (95 % CI)	*P* value	OR (95 % CI)	*P* value
Lowest	1 (reference)		1 (reference)		1 (reference)		1 (reference)	
Mid	2.36 (1.370–4.078)	0.002	2.28 (1.313–3.967)	0.003	1.71 (0.729–3.991)	0.219	1.63 (0.674–3.924)	0.279
Highest	2.50 (1.432–4.348)	0.001	2.54 (1.418–4.544)	0.002	3.42 (1.519–7.709)	0.003	3.19 (1.371–7.424)	0.007
P for trend	0.002		0.002		0.002		0.006	

Model 1: adjusted for age and sex

Model 2: model + adjusted for HbA1c and duration of diabetes

Model 3: model 2 + adjusted for systolic blood pressure, LDL cholesterol, estimated GFR, uric acid and smoking

Model 4: model 3 + adjusted for insulin, oral hypoglycemic agents, antiplatelet agents, antihypertensive medication, and statin

*CI* confidence interval, *GFR* glomerular filtration rate, *OR* odds ratio

## Discussion

In the present study, we observed that the TyG index was correlated with HOMA-IR and that there were significant differences in cardiometabolic parameters, including the presence of metabolic syndrome, according to the TyG index. In addition, we demonstrated that the higher TyG index is associated with an increased risk of CAS in asymptomatic subjects with type 2 diabetes. Furthermore, subgroup analysis showed that the TyG index is significantly associated with CAS in subjects with cardiovascular risk factors such as older age, longer duration of diabetes, poor glycemic control, hypertension, absence of statin or antiplatelet agents use, and male gender. This suggests that the TyG index may be used as a marker for insulin resistance and, importantly, to help identify subjects at high risk of CVD.

Insulin resistance is a hallmark of type 2 diabetes and even precedes the disease for several years [14]. Many studies indicate that insulin resistance per se and associated disorders contribute to the development of CVD in nondiabetic as well as diabetic subjects. It is well known that insulin resistance is related to cardiometabolic risk factors, such as hyperglycemia, dyslipidemia, and hypertension. In addition, inflammatory and pro-coagulant properties of insulin resistance may also lead to accelerated atherosclerosis [14, 15]. For these reasons, insulin resistance has been considered as not only a pathogenic cause but also a predictor of CVD in type 2 diabetes. Several prospective studies have shown that insulin resistance predicts incident CVD in both the general and diabetic subjects [5, 7, 16, 17]. In the general population, insulin resistance, presented as HOMA-IR or metabolic syndrome, was an independent predictor of CVD after adjusting for other risk factors [7, 16, 17]. Similar results were observed in subjects with type 2 diabetes and a hazard ratio for CVD was significantly greater in subjects with diabetes compared with those without diabetes [5, 17].

In accordance with previous studies, we observed that the TyG index, a marker for insulin resistance, was significantly associated with CAS in asymptomatic subjects with type 2 diabetes. The TyG index was first introduced as a surrogate marker for insulin resistance by Guerreo-Romero [9]. The TyG index has shown direct correlation with insulin resistance, as assessed by hyperinsulinemic euglycemic clamp or insulin-mediated glucose uptake [18, 19]. In a Brazilian population, TyG index showed better performance for assessing insulin resistance than the HOMA-IR [20]. The TyG index showed similar correlation with insulin resistance regardless of sex, obesity, and diabetes [18]. This suggests that the TyG index might be used in various populations as a biomarker of insulin resistance. In addition, previous studies have revealed that the TyG index has an independent association with incident diabetes and carotid atherosclerosis [20–22]. In the present study, we observed that the TyG index was correlated with HOMA-IR and associated with an increased prevalence of metabolic syndrome. Taken together, the TyG index might be useful for the identification of subjects with insulin resistance and associated disorders.

Several potential mechanisms have been suggested to explain the correlation between TyG index and insulin resistance. Increased triglyceride levels can lead to elevated free fatty acids and, thus, increased flux of free fatty acids from adipose to nonadipose tissue, which may induce insulin resistance [23, 24]. Previous studies have demonstrated that higher levels of triglycerides in the liver and muscle may interfere with glucose metabolism in each target organ [25–27]. These findings support the importance of triglycerides in the pathogenesis of insulin resistance and its possibility to be used as a surrogate marker for insulin resistance. Recently, there have been growing evidence that some nutraceuticals in common diet lower plasma lipid levels and, thus, reduce the overall cardiovascular risk [28]. In terms of TyG index, it might be postulated that the effects of these nutraceuticals on cardiovascular risk would be mediated by improving insulin resistance.

Of note, the association between TyG index and CAS was more significant in subjects with cardiovascular risk factors such as older age, longer duration of diabetes, poor glycemic control, lack of antiplatelet agent or statin use, and male gender. This implies that greater insulin resistance, as represented by the TyG index, makes subjects more vulnerable to risk factors related to CVD. Consistent with our results, high triglyceride levels has been reported to be a predictor of incident or recurrent CVD in subjects with type 2 diabetes [2, 29]. To prevent CVD, aggressive management of modifiable risk factors such as hyperglycemia, hypertension, or dyslipidemia in subjects with cardiovascular risk factors and high TyG index may be necessary. In contrast to other risk factors, the association between TyG index and CAS was less significant in subjects with hypertension. In our study, more than 50 % of subjects with hypertension had taken angiotensin receptor blocker or beta blocker. This may contribute to the different results in subjects with hypertension.

This study has several limitations. Firstly, as this study has cross-sectional observation study design, we could not evaluate for a direct causal relationship. Secondly, we compared the TyG index with HOMA-IR rather than the hyperinsulinemic euglycemic clamp, a gold standard method for measuring insulin resistance. However, previous studies have shown similar associations between the TyG index and insulin resistance, as measured by the hyperinsulinemic euglycemic clamp, in a wide range of subjects [18, 20]. Thirdly, the TyG index may not be used in subjects with extremely high triglyceride levels or in those taking medications that lower mainly triglycerides. As more than 50 % of the participants had taken statins, we only excluded subjects taking fenofibrate or omega-3. Lastly, we could not adjust for alcohol, fruits, juices, and other simple sugars consumption, which can affect blood triglyceride levels. However, the measurement of CAS by coronary CT angiography, as opposed to surrogate markers such as intima-media thickness, is a strength of this study. In addition, we analyzed data with a relatively large sample size of asymptomatic subjects with type 2 diabetes. One of the advantages of the TyG index is that both the triglyceride and glucose are usually measured in routine clinical practice and is, therefore, readily available. The TyG index can also be obtained by a simple equation and is less costly than the insulin measurement.

## Conclusions

In conclusion, the TyG index is associated with an increased risk of CAS in asymptomatic subjects with type 2 diabetes, particularly when they have other risk factors. The TyG index may be used as a marker for insulin resistance and help identify subjects at high risk of CVD in asymptomatic subjects with type 2 diabetes. Further prospective studies are needed to investigate whether TyG index can predict cardiovascular events in these subjects.

## Abbreviations

ACR: Albumin to creatinine ratio
ANOVA: Analysis of variance
BMI: Body mass index
CAS: Coronary artery stenosis
CI: Confidence interval
CRONOS-ADM: Coronary CT angiography evaluation for clinical outcome in asymptomatic patients with type 2 diabetes
CT: Computed tomography
CVD: Cardiovascular disease
DBP: Diastolic blood pressure
DPP-IV: Dipeptidyl peptidase IV
ECG: Electrocardiography
FPG: Fasting plasma glucose
GFR: Glomerular filtration rate
HDL: High density lipoprotein
HOMA-IR: Homeostasis model assessment of insulin resistance
LDL: Low density lipoprotein
NCEP-ATP III: National cholesterol education program adult treatment panel III
OR: odds ratio
PPG: Postprandial plasma glucose
SBP: Systolic blood pressure
TyG index: Triglyceride-to-glucose index
WC: Waist circumference
